# Commentary on: Use of Remifentanil and Alfentanil in Endotracheal Intubation: A Comparative Study

**DOI:** 10.5812/aapm.3643

**Published:** 2012-04-01

**Authors:** Mahdi Najafi

**Affiliations:** 1 Tehran Heart Center, Tehran University of Medical Sciences (TUMS), Tehran, Iran

**Keywords:** Alfentanil, Remifentanil, Intubation, Endotracheal

Dear Editor, 

I read the paper by Imani et al. (1) with interest and must congratulate them in attempting to show the advantages of administering remifentanil for laryngoscopy and tracheal intubation in adult patients. It was interesting to know the experiences of colleagues with regard to tracheal intubation in noncardiac patients, as I use a combination of remifentanil and propofol in my daily practice in cardiac anesthesia. The primary goal of this study was to evaluate patients’ conditions during tracheal intubation after remifentanil compared with alfentanil. Although the scoring criteria were well defined, the question arises as to why the authors did not report the hemodynamic situation of the patients before and after induction of anesthesia, as other studies have done (2-5). Their report did not describe whether the patients had bradycardia or hypotension. We know that hemodynamic instability is a common feature of remifentanil, alfentanyl, and propofol, leading some researchers to use atropine as prophylaxis to overcome decreases in heart rate after induction with these drugs (2, 3). 

The other question is the time from injection to apnea or respiratory depression, if applicable. It would be helpful for colleagues to know this interval and the incidence of apnea in the remifentanil and alfentanil groups, as we already know that the incidence of apnea is high with opioids, especially in combination with other anesthetics, such as propofol. It is noteworthy that the rate and severity of respiratory depression with different dosages and combinations of anesthetic and analgesics are controversial (6). It appears from the study that there was no significant difference between the remifentanil and alfentanil groups scores regarding intubation condition rather than vocal cord patency score; however, the authors should clarify if there is any difference between genders and age groups.
